# Endogenous Expression of an RNA-Hydrolyzing Minibody Enhances the Therapeutic Effects Against Influenza a Virus Compared to Therapeutically Intranasal Administration

**DOI:** 10.3390/ijms27177850

**Published:** 2026-09-02

**Authors:** Quynh Xuan Thi Luong, Yongjun Lee, Chengmin Lin, Muhammad Salman Akram, Phuong Thi Ho, Phuong Thi Hoang, Ramadhani Qurrota Ayun, Thuy Thi Bich Vo, Taek-Kyun Lee, Sukchan Lee

**Affiliations:** 1 Faculty of Pharmacy, Ton Duc Thang University, Ho Chi Minh City 70000, Vietnam; luongthixuanquynh@tdtu.edu.vn; 2 Department of Integrative Biotechnology, Sungkyunkwan University, Suwon 16419, Republic of Korea; 88yjl11@naver.com (Y.L.); lincm921@naver.com (C.L.); salmanakram303@gmail.com (M.S.A.); hophuongk59sinhhoc@gmail.com (P.T.H.); hoangphuong06cs@gmail.com (P.T.H.); ramadhani.qurrota@gmail.com (R.Q.A.); 3 Faculty of Biotechnology, Ho Chi Minh City Open University, Ho Chi Minh City 70000, Vietnam; thuy.vtb@ou.edu.vn; 4 Ecological Risk Research Department, Korea Institute of Ocean Science & Technology, Geoje 53201, Republic of Korea

**Keywords:** 3D8 scFv, RNA-hydrolyzing minibody, transgenic mice, influenza virus, antiviral agent

## Abstract

Influenza pandemics have had devastating impacts in the 20th and 21st centuries, resulting in millions of deaths. It is challenging to control the influenza virus through antiviral drugs due to its rapid evolution to evade the host immune system. The 3D8 single chain variable fragment (scFv) protein, known for its nuclease activity against DNA and RNA viruses, has demonstrated broad antiviral properties in various models. In this study, we investigated the impact of administration routes and dosages on the distribution of exogenously administered 3D8 scFv. Our results suggested that repeated intranasal (IN) injection primarily targets lung tissue, while intravenous (IV) administration ensures systemic distribution, including lung tissues via the bloodstream. Furthermore, we explored the efficacy of exogenous and endogenous 3D8 scFv in inhibiting influenza A virus (H1N1/PR8) replication in mouse models. Our findings revealed that while both approaches exhibited antiviral activity, with constitutive pre-infection expression of 3D8 scFv showed 100% survival, whereas mice receiving exogenous 3D8 scFv post-infection exhibited 33.3% survival. These findings highlight the potential of 3D8 scFv as a promising antiviral strategy against influenza and suggest its possible applications in transgenic livestock for future disease control.

## 1. Introduction

Respiratory viral infections present significant global health challenges, causing substantial morbidity and mortality, particularly among vulnerable populations [1,2,3,4]. The 20th century witnessed three influenza pandemics in 1918 (H1N1), 1957 (H2N2), and 1968 (H3N2), which resulted in 50–100 million, 1.1 million, and 1 million deaths, respectively, worldwide [4]. The first influenza pandemic of the 21st century emerged with the H1N1/pdm09 virus in 2009, resulting in approximately 151,700–575,400 deaths in its first year [5,6]. Annually, seasonal influenza affects billions globally, with 3–5 million cases of severe disease and 290,000–650,000 respiratory fatalities, predominantly among children under 5 years of age in developing countries [7]. During the COVID-19 pandemic, influenza prevalence was notably low due to social distancing, mask usage, lockdowns, and quarantines [8]. However, as these nonpharmaceutical interventions were lifted, influenza resurged in late 2021, starting with early seasonal activity in the Southern Hemisphere during the first half of 2022 and continuing with unusually late and extended seasons in the Northern Hemisphere [8,9]. To control and mitigate influenza, numerous antiviral drugs have been developed, targeting specific sites on the virus at different stages of its life cycle. Food and Drug Administration (FDA)-approved antiviral drugs include Rimantadine and Amantadine, which target the M2 ion channel, and neuraminidase (NA) inhibitors such as Oseltamivir, Zanamivir, Peramivir, and Baloxavir Marboxil, which inhibit polymerase acidic protein (PA) [6,10]. However, influenza viruses evolve continuously, often showing reduced susceptibility to existing antiviral drugs, presenting ongoing challenges for healthcare providers, particularly when new viral variants or strains emerge, and available antiviral treatments become ineffective [6,11].

A monoclonal antibody (mAb) named 3D8 was originally identified and isolated as an anti-DNA antibody from the spleen cells of MRL-lpr/lpr mice, an autoimmune-prone model that closely mimics human systemic lupus erythematosus. Subsequently, the 3D8 single chain variable fragment (scFv) is engineered as a recombinant protein by linking a variable heavy chain with a variable light chain via a flexible polypeptide (Glycine4-Serine1)3 linker [12,13,14]. This protein exhibits Mg^2+^-dependent nuclease activity and can hydrolyze both DNA and RNA without sequence specificity [13]. The antiviral properties of 3D8 scFv have been demonstrated against various DNA and RNA viruses in both in vitro and in vivo models [10,11,15,16,17,18]. Additionally, transgenic (TG) animals harboring the 3D8 scFv gene have been developed to combat pseudorabies virus (TG mice), avian influenza virus (TG chicken), and bronchitis virus (TG chicken) [16,19,20]. In this study, we investigated the impact of administration routes and dosages on the distribution of exogenous 3D8 scFv in various organs, including lung, liver, kidney. We also assessed the inhibition of influenza A virus (H1N1/PR8) replication by using exogenous 3D8 scFv as a therapeutic approach and endogenous 3D8 scFv as TG mouse models. Our results showed that endogenous 3D8 scFv expression in TG mice displayed significant antiviral activity against the influenza virus.

## 2. Results

### 2.1. Impact of Administration Route and Dosage on the Organ Distribution of 3D8 scFv

**Figure 1:**
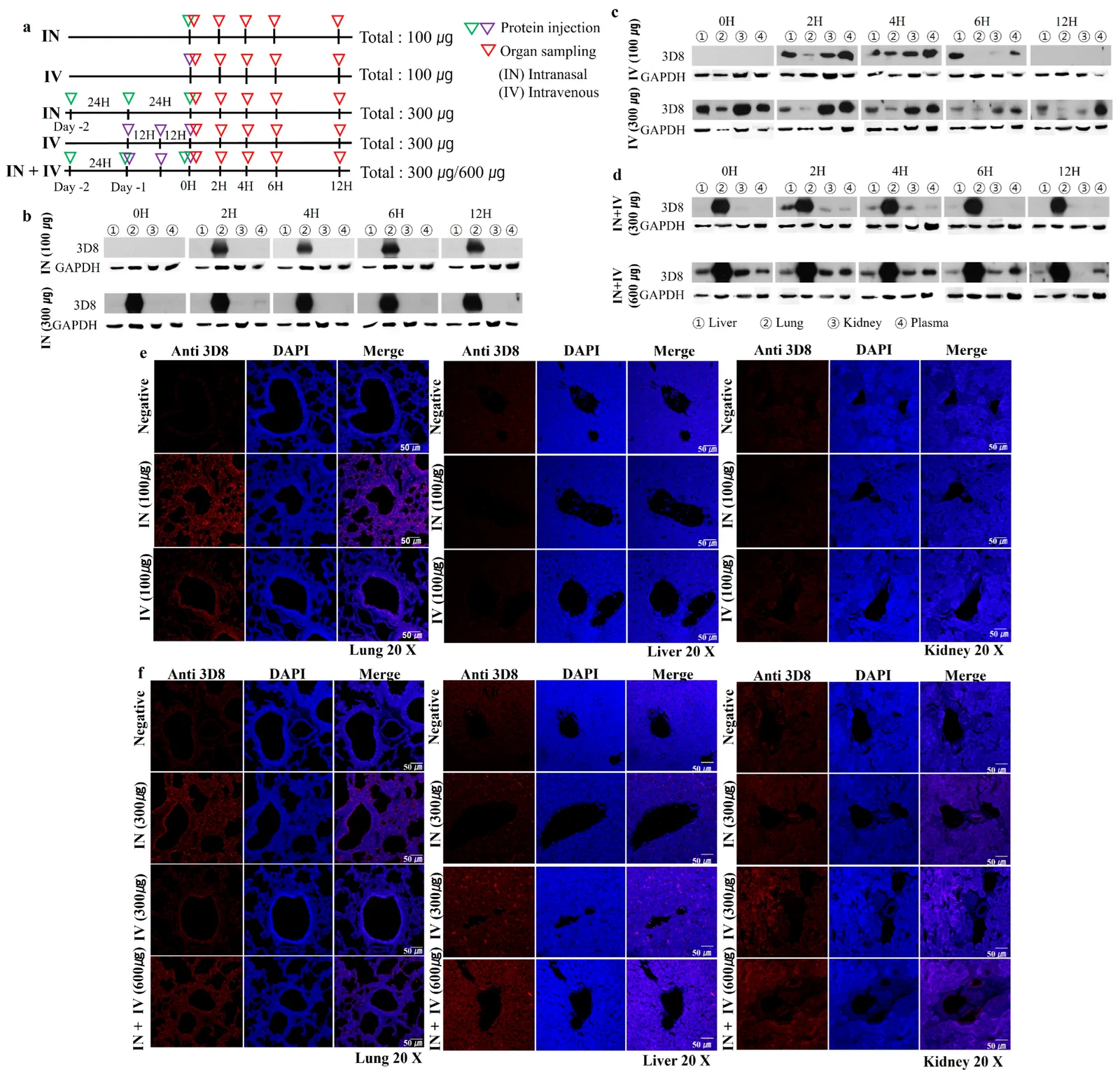
Impact of administration route and dosage on the organ distribution of 3D8 single chain variable fragment (scFv). (**a**) Schematic diagram for 3D8 scFv treatment plan. 3D8 scFv was administered via IN, IV (single route), and IN + IV (combined routes) into mice once or thrice (100 μg/injection). IN and IV injections were administered thrice at 24 h and 12 h intervals, respectively. The combined IN + IV groups received 100 μg/injection (total 600 μg) or 50 μg/injection (total 300 μg). Mouse organs and plasma were sampled at 0, 2, 4, 6, and 12 h periods. (**b**–**d**) The retention time of 3D8 scFv was measured in mice organs and plasma, with the numbers indicating ① liver, ② lung, ③ kidney, and ④ plasma. Western blot analysis using rabbit anti-3D8 antibody demonstrated the presence of 3D8 scFv in mice. (**e**,**f**) Immunohistochemistry results showing the penetration of 3D8 scFv in different organs. Four hours after perfusion, 3D8 scFv was found to be localized in the lung, liver, and kidney tissues. Nuclei were detected by DAPI staining (blue), and 3D8 scFv was visualized with TRITC (red). Scale bar: 50 μm.

The 3D8 scFv was successfully expressed and purified, and its purity was assessed using the Coomassie blue staining method. The characteristics of the purified 3D8 scFv were analyzed and are presented in Figure S1. Sequentially, the impact of administration route and dosage on the organ distribution of 3D8 scFv was investigated using intranasal (IN), intravenous (IV), and combined (IN + IV) injections. Mice were administered 100 μg of 3D8 scFv per dose. The organs (liver, lungs, and kidneys) and plasma samples were collected at 0, 2, 4, 6, and 12 h and 3D8 scFv protein and GAPDH were analyzed using Western blotting (Figure 1a). For single-dose administration, 3D8 scFv was detected in lung tissues from 2 to 12 h following IN administration, but not in other organs (Figure 1b). In contrast, after IV injections, 3D8 scFv was detected in the liver, lungs, kidneys, and plasma at 2 and 4 h, but only in the liver and plasma at 6 h, indicating a shorter retention time compared to IN administration (Figure 1c). Multiple doses of 3D8 scFv were administered to extend the retention time. IN injection was given every 24 h, IV every 12 h, and combined IN + IV (100 μg or 50 μg per injection) (Figure 1a). And these treatments were administered prior to organs sampling. Repeated IN administration resulted in higher 3D8 scFv levels in the lungs, while IV injection extended retention in all organs up to 12 h (Figure 1b,c). The 3D8 scFv levels in lung tissues, detected by Western blot analysis (Figure 1d), following combined IN + IV administration were comparable to those observed with IN administration alone, as shown in Figure 1b.

In another experiment, following blood perfusion, lung tissue sections revealed strong 3D8 scFv penetration after a single-dose administration via the IN route but less via the IV route. However, 3D8 scFv was not detected in the liver and kidneys across all injection routes (Figure 1e). Further analysis of triple-dose administration revealed that, after IN injection, 3D8 scFv localized primarily in the lungs, with small amounts detected in the liver and kidneys (Figure 1f). IV injection with triple dose resulted in a broader distribution, with less 3D8 scFv detected in the lungs compared to other organs (Figure 1f). Combined IN + IV administration produced results comparable to those observed with individual routes, showing strong 3D8 scFv signals in the lungs (IN-100 µg, IN-300 µg), liver (IV-300 µg), and kidneys (IV-300 µg) (Figure 1f). This is consistent with the Western blot results, which showed that 3D8 scFv levels were highest in the lungs following IN and IN + IV administration, whereas stronger signals were observed in the liver and kidney tissues after IV administration (Figure 1b–f).

These results suggested that repeated IN injection primarily targets lung tissues, while IV administration ensures systemic distribution, including lung tissues via the bloodstream.

### 2.2. Production of Transgenic Mice Expressing 3D8 scFv

**Figure 2:**
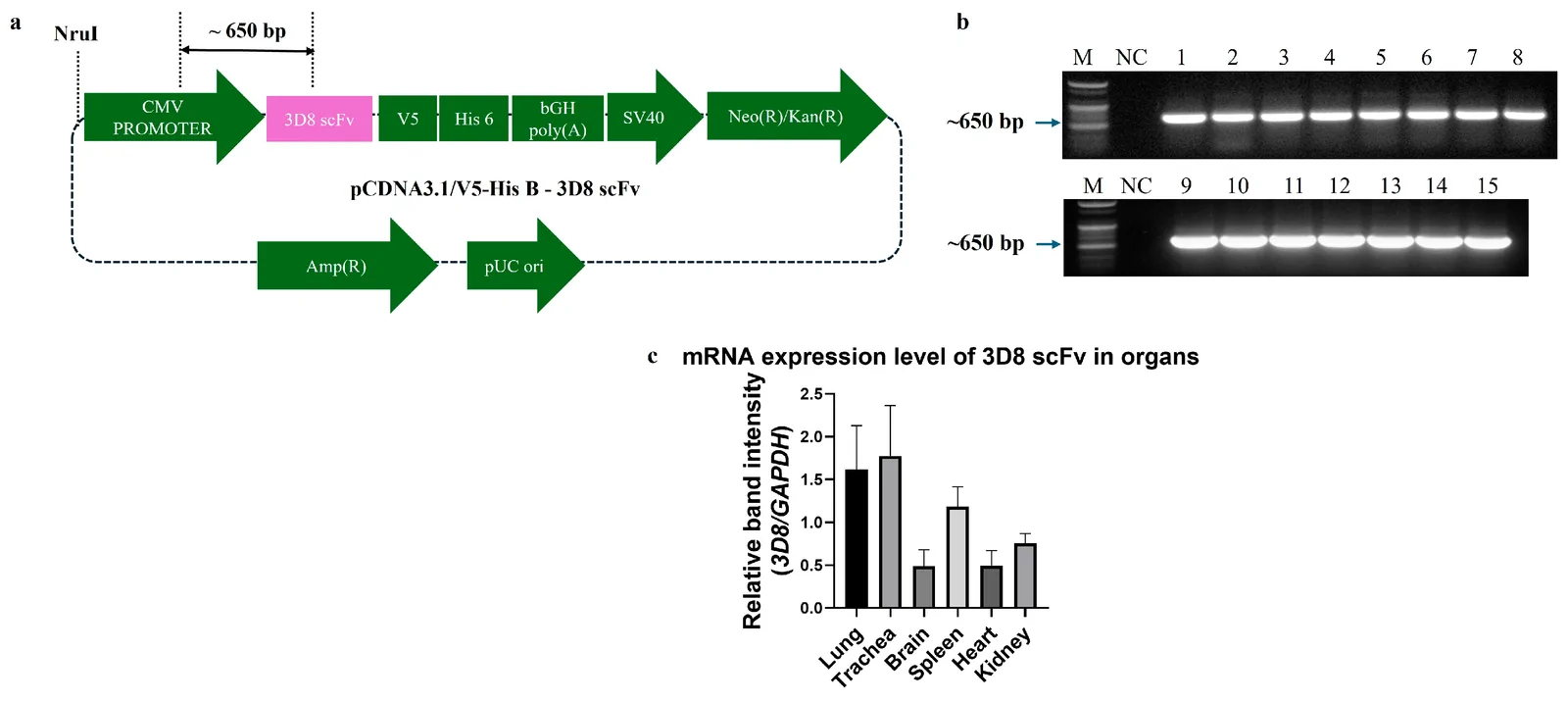
Generation of transgenic (TG) mice models harboring the 3D8 scFv gene. (**a**) Schematic diagram of the 3D8 scFv expression cassette. The pink region represents the 3D8 scFv gene, while the green regions represent the corresponding components of the pCDNA3.1/V5-HisB plasmid. (**b**) The presence of the 3D8 scFv gene (650 bp) was confirmed in TG mice using specific primers (please refer to Section 4). NC indicates negative control, number 1-15 represents 15 TG mice analyzed. Then, they were applied for antiviral experiments. M: DNA molecular weight marker, NC: C57BL/6N gDNA. (**c**) RT-PCR analysis of 3D8 scFv mRNA in TG mice tissues and the relative band intensity was calculated after normalizing the intensity of the 3D8 scFv bands to that of GAPDH using ImageJ2. 3D8 scFv mRNA was detected in the lungs, trachea, brain, spleen, heart, and kidneys.

The pcDNA3.1-3D8 scFv plasmid (outlined in Figure 2a) was linearized with the NruI enzyme and was introduced into C57BL/6NCrjBgi mice to establish the 3D8 scFv transgenic (TG) mice. After transformation, all resulting TG individuals were subjected to PCR analysis at Macrogen Co. Following this, ten female and three male TG mice were chosen for breeding to generate offspring. The presence of the 3D8 scFv gene in 15 mice was confirmed via PCR, evidenced by bands approximately ~650 bp in size (Figure 2b). These TG mice were employed for further investigations. Furthermore, the expression profiles of 3D8 scFv across various organs, including the lungs, trachea, brain, spleen, heart, and kidneys, were evaluated using RT-PCR in TG mice and wild-type (WT) mice. The findings revealed that 3D8 scFv was ubiquitously expressed across all tissues in the TG mice (Figure 2c), with notably higher mRNA expression levels in the lungs, trachea, and spleen compared to other tissues (Figure 2c), In contrast, no 3D8 scFv expression was detected in WT mice.

### 2.3. Effect of Exogenous and Endogenous 3D8 scFv on the Virulence of the Influenza Virus in the Lung

First, the safety of exogenous 3D8 scFv in mice was examined. C57BL/6 mice were divided into six cohorts: IN-Neg, IN-100, IN-200, IV-Neg, IV-100, and IV-200. These cohorts underwent IN or IV administrations with DPBS or 3D8 scFv at dosages of 100 µg and 200 µg, respectively (Figure S2a). Following a 14-day monitoring period, 3D8 scFv did not exert any discernible effects on mice behavior, encompassing several parameters (Figure S2b). No significant reductions in body weight were noted across any group during the experiment, and no deaths were recorded (Figure S2c). Furthermore, serum biochemical analyses were performed alongside clinical assessments to evaluate potential organ impairments by quantifying surrogate markers in bodily fluids. Most serum biochemical parameters remained within the normal reference range across all cohorts (Table S2).

**Figure 3:**
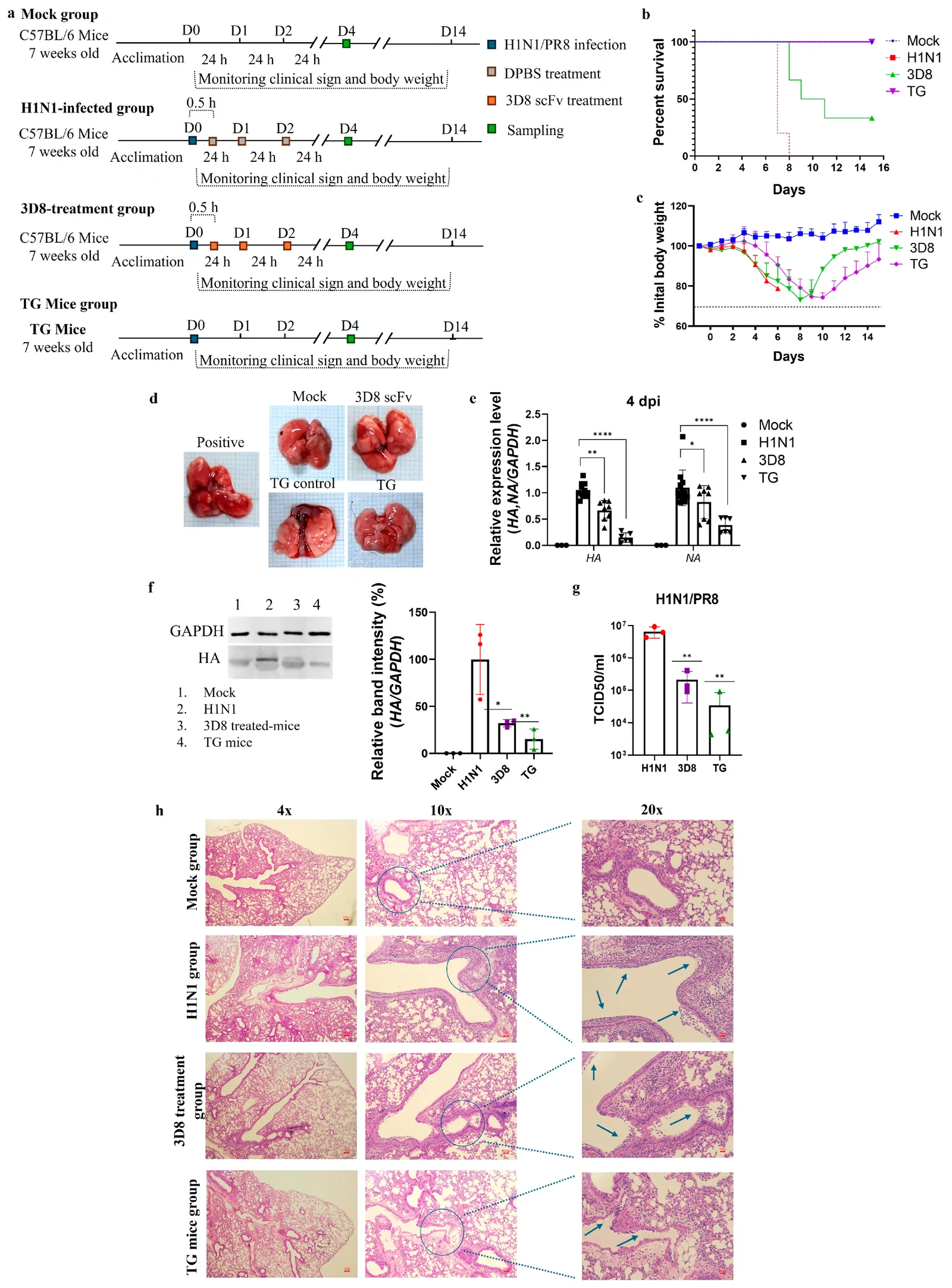
Evaluating survival rate and body weight of the 3D8-treated and TG groups. (**a**) Schematic diagram of experimental design. C57BL/6 and TG mice (7–8 weeks old) were infected with H1N1/PR8 on day 0. Then, mice in the 3D8 scFv treatment group were administered 100 µg 3D8 scFv intranasally at 0.5 h post-infection and subsequently at 24 h intervals on days 1 and 2, for a total of three administrations and a cumulative dose of 300 µg per mouse. On day 4, all groups were euthanized, and their lungs were harvested for further experiments. (**b**) Survival rate and (**c**) body weight of mice was monitored for 14 days post-infection (dpi). (**d**) The impact of influenza virus on lungs in the presence of 3D8 scFv. Gross pathology of mice lungs was collected at 4 dpi. (**e**) To evaluate the antiviral effect of 3D8 scFv, the relative expression levels of influenza viral genes (HA and NA) were measured by RT-qPCR using mRNA extracted from the mice lungs. (**f**) The hemagglutinin (HA) protein levels of IAVs were tested and calculated based on the percentage of the band intensity after normalizing it with that of GAPDH. (**g**) Viral load was calculated based on TCID_50_/mL. (**h**) Lung histopathology of noninfected and infected, 3D8-treated, and TG mice was analyzed. Cell debris and inflammatory cells in bronchiolar lumen (arrow). The data are presented as mean ± SD. Statistical significance was determined by two-way ANOVA (* *p* < 0.05, ** *p* < 0.01, **** *p* < 0.0001).

To evaluate the antiviral efficacy of exogenously administered and endogenously expressed 3D8 scFv against influenza infection, four distinct cohorts of seven-week-old C57BL/6 WT and TG mice were established: a nontreatment, nonvirus-infected (mock), and three treatment groups (administered with 2LD_50_ of H1N1/PR8, 750 PFU). The three treatment cohorts comprised a DPBS-treated (H1N1-infected) group, a group treated with 100 µg of 3D8 scFv administered once daily for three consecutive days (3D8-treated group; treatment initiated at 0.5 h post-infection on day 0, followed by administration on days 1 and 2 at 24 h intervals) via IN administration, and TG mice expressing endogenous 3D8 scFv (TG group) (Figure 3a). Clinical parameters, encompassing survival rates and body weight, were monitored for 14 days post-infection. By day 8 post-infection, while the mortality rate in the H1N1-infected group reached 100% (5/5), 33.3% and 100% of the mice in the 3D8-treated (2/6) and TG groups (5/5) survived, respectively, until 14 dpi (Figure 3b,c). Regarding body weight, the surviving mice in the 3D8-treated group displayed a more rapid recovery than the TG group (Figure 3c).

To assess the antiviral efficacy of 3D8 scFv following H1N1/PR8 infection, lung samples were collected from mice at 4 dpi. Unlike the virus-infected WT mice, the lungs of the virus-challenged TG and 3D8-treated groups did not display any dark red discoloration (Figure 3d). RT-qPCR revealed significant downregulation of HA and NA mRNA expression in both treatment groups, with reductions of approximately 30% (HA) and 25% (NA) in 3D8-treated mice, and 85% (HA) and 62% (NA) in TG mice, relative to the H1N1-infected positive control group (Figure 3e).

Additionally, viral protein expression level was significantly reduced as less than 40% protein was detected in the 3D8-treated and TG groups (Figure 3f). Viral load was reduced in the presence of 3D8 scFv, which was approximately 30-fold and nearly 200-fold lower in 3D8-treated and TG groups, respectively, compared to the H1N1-infected group (Figure 3g). At 4 dpi, histopathological analysis revealed that H1N1/PR8 infection induced significant cellular degeneration and necrosis, with cellular debris in the bronchiolar lumen. Further, extensive inflammation was observed, marked by the infiltration of inflammatory cells, including macrophages and neutrophils (Figure 3h). In 3D8-treated and TG mice, the cellular debris was notably reduced compared to the H1N1-infected group, although inflammatory cells were still present in the lung tissues. Furthermore, the alveolar air sacs were better preserved in 3D8-treated mice than in virus-infected mice (Figure 3h).

### 2.4. The Recovery of 3D8-Treated and TG Mice After 20 Days H1N1/PR8 Challenge

**Figure 4:**
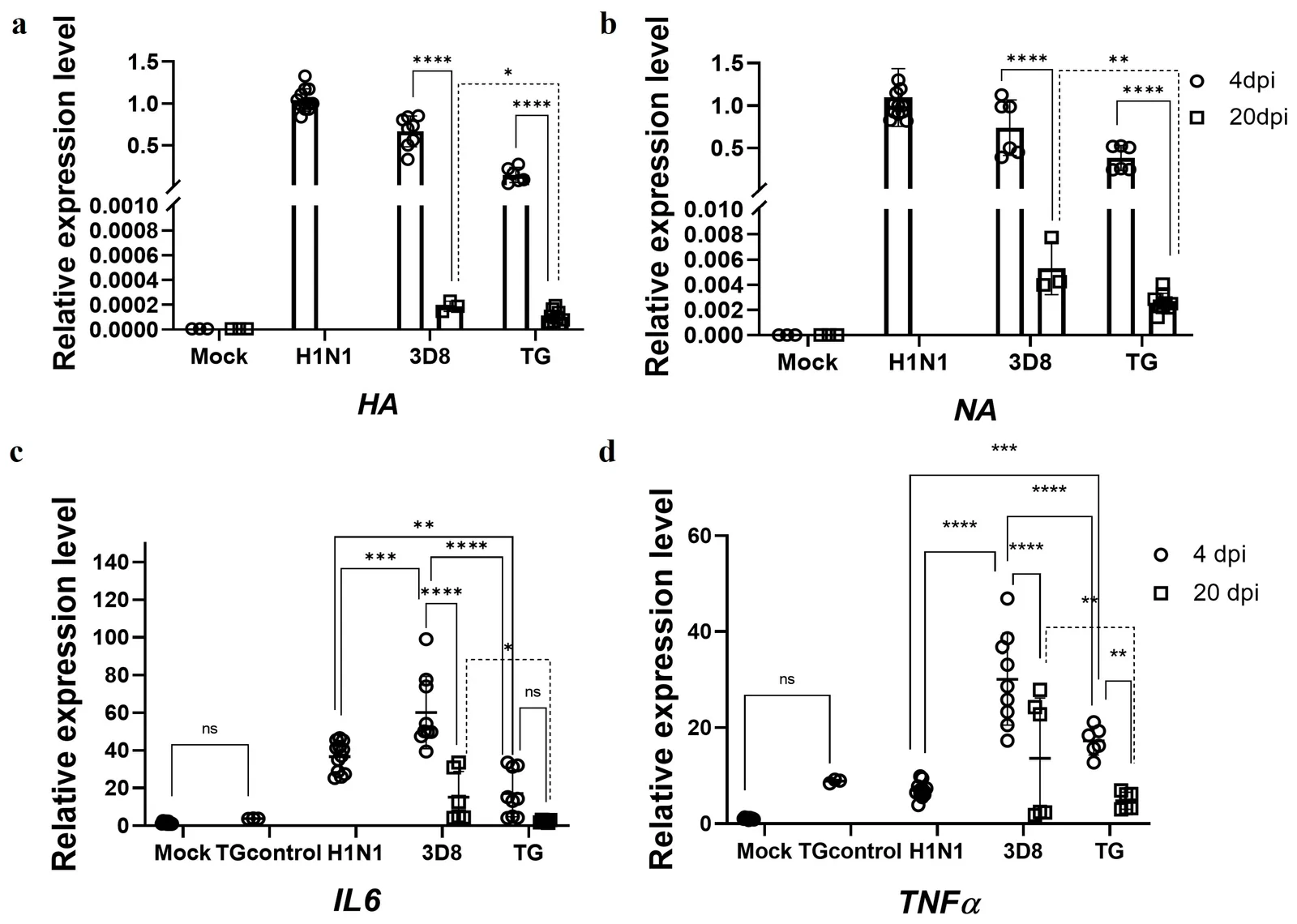
3D8 scFv treatment group and TG mice recovery after influenza virus challenge. Expression levels of the (**a**) HA and (**b**) NA genes of the influenza virus in the lungs were analyzed by RT-qPCR at 20 dpi and compared to those at 4 dpi. (**c**,**d**) Cytokines (IL-6 and TNF-α) gene expression levels were analyzed by RT-qPCR in mice of all groups (mock, TG control, H1N1-infected, 3D8, TG) at 4 dpi and 20 dpi. The data are presented as mean ± SEM. Statistical significance was determined by one-way ANOVA (* *p* < 0.05, ** *p* < 0.01, *** *p* < 0.001, **** *p* < 0.0001; ns, not significant).

The body weights of the remaining 3D8 scFv-treated and TG mice were continuously monitored to evaluate their recovery efficacy. To assess the persistence of viral RNA following infection, HA and NA gene expression levels were evaluated at 20 dpi. Less than 0.02% of HA gene expression was detected at 20 dpi, compared to approximately 70% at 4 dpi in both 3D8-treated and TG mice (Figure 4a). Similarly, NA gene expression was reduced to 0.2% in both groups at 20 dpi compared to the levels observed at 4 dpi (Figure 4b).

Regarding cytokine responses, the IL-6 and TNF-α gene expression levels were significantly upregulated following influenza virus infection by approximately 25-fold and seven-fold, respectively, in the H1N1-infected group compared to the mock group at 4 dpi (Figure 4c,d). In the 3D8 scFv-treated group, IL-6 expression was 1.5 times higher than in the H1N1-infected group. In contrast, it was 0.5 times lower in the TG mice compared to the H1N1-infected group (Figure 4c). Conversely, TNF-α expression was 4.5 and 2.5 times higher in the 3D8 scFv-treated and TG mice, respectively, compared to the H1N1-infected group (Figure 4d). By 20 dpi, as the viral load decreased, IL-6 levels dropped four-fold in the 3D8 scFv-treated group compared to day 4, and in the TG group, IL-6 levels reached values comparable to the mock (Figure 4c). Moreover, TNF-α levels decreased 2.2-fold in the 3D8 scFv-treated group and 3.6-fold in the TG group by day 20 relative to day 4 (Figure 4d).

## 3. Discussion

Numerous studies have demonstrated the antiviral efficacy of 3D8 scFv against several DNA and RNA viruses both in vitro and in vivo, underscoring its potential as a broad-spectrum antiviral agent [10,11,15,18]. Based on these findings, 3D8 scFv was utilized as a therapeutic approach to protect C57BL/6 mice from H1N1/PR8. In vivo IN administration of 3D8 scFv following viral challenge enhanced survival rates and reduced body weight loss (Figure 3b,c). Specifically, 33.3% of mice survived the H1N1/PR8 infection. This is consistent with a previous study that reported a 40% survival rate in mice challenged with H1N1/pdm09 and treated with 50 µg 3D8 scFv per dose, administered over four doses. However, the differences between the studies were not statistically significant, which could be attributed to the differences in mouse strains, gender, or 3D8 scFv dosages [10].

The survival rate was significantly higher when mice were pretreated with 3D8 scFv against influenza A virus [10,17]. Further, histopathological analysis of the lung tissues from mice challenged with H1N1/PR8 displayed typical features of lung damage, including infiltration of inflammatory cells, and cell debris sloughing in the bronchiolar lumen, as shown in previous studies [10,21,22,23]. However, the lung lesions were less severe in the 3D8-treated group compared to the PBS-treated group, consistent with Yongjun et al., 2022 [10]. In addition, while H1N1/PR8 infection was lethal in the C57BL/6 WT mice, the TG mice expressing 3D8 scFv mRNA in various tissues (lungs, trachea, brain, spleen, kidneys, heart, and liver) showed 100% survival, while the 3D8-treated group had 33.3% survival (Figure 3b,c).

**Figure 5:**
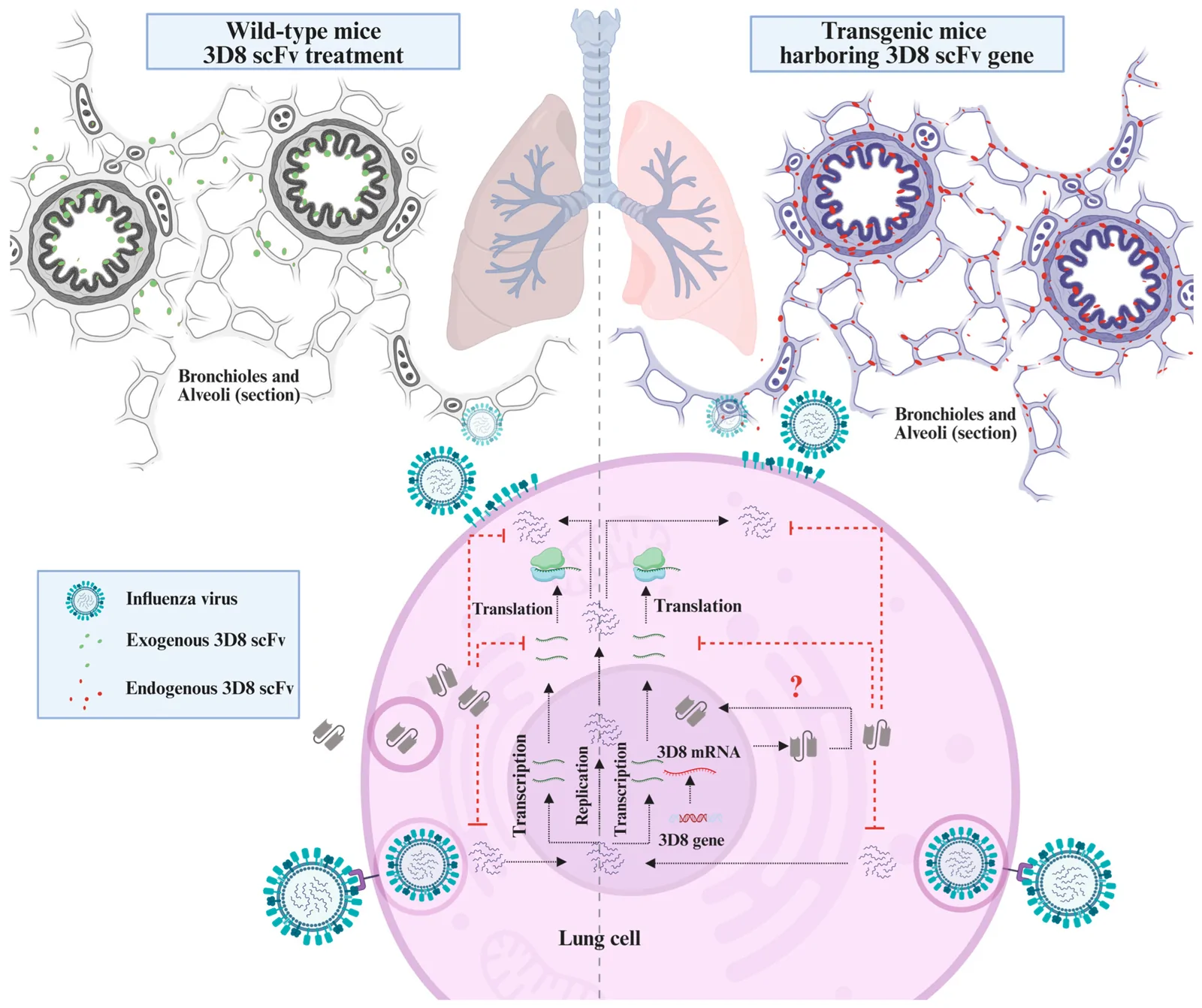
Illustration of the proposed mechanism of action of exogenous and endogenous 3D8 scFv against influenza virus in vivo. Left Panel: After being administered intranasally into mice, 3D8 scFv traversed the respiratory tract, reaching the trachea, bronchioles, and alveoli, where it was distributed to the epithelial cells lining the respiratory tract. However, the concentration of exogenously administered 3D8 scFv gradually decreased over time due to factors such as mucociliary clearance and metabolic degradation. Right Panel: Endogenously expressed 3D8 scFv was present in every cell within the lung as the 3D8 gene was integrated into the mouse genome. This genomic integration ensured continuous expression of 3D8 scFv in lung cells. Although there is no direct evidence of 3D8 scFv translocating to the nucleus in this context, previous studies on transgenic cell lines have demonstrated that 3D8 scFv is localized in both cytosol and nucleus.

Moreover, the viral mRNA expression levels (HA and NA) were significantly lower in the TG mice than the 3D8-treated group, suggesting that even a small amount of intrinsic 3D8 scFv, undetectable by Western blot, can protect mice from viral inoculation. This protective ability of intrinsic 3D8 scFv expression in TG animals has been previously demonstrated: 56% of TG mice survived PRV intramuscular infection, and TG chicken expressing 3D8 scFv exhibited antiviral effects against H9N2 infection, bronchitis virus transmission, and survived aerosol exposure to the Newcastle disease virus [15,19,20,24]. This indicates that low concentrations of 3D8 scFv expressed in TG mice can confer antiviral effects without harming the host. Conversely, IN administration of drugs has several challenges, such as the impact of nasal secretions, ciliary movement, and nasal vascularity on drug absorption rates. Moreover, the metabolism of drugs in the nasal cavity by enzymes can alter their absorption, especially for proteins and peptides [25,26,27]. Conditions like the common cold can impair absorption due to mucosal membrane changes, and the mucociliary clearance mechanism might also be involved. Additionally, the previous data performed that the retention time of 3D8 scFv can be a limitation, as its levels gradually diminish over time, with near complete clearance observed by three days post-treatment [10]. Administering high protein concentrations can result in a viscous solution, which may impede inhalation in mice, potentially inducing stress and confounding experimental outcomes. Several studies have demonstrated that intranasal administration facilitates both systemic and nose-to-brain drug delivery, owing to the extensive vasculature and olfactory nerves in the nasal cavity, enabling transport across the blood–brain barrier [26,27,28]. Additionally, research on the distribution of intranasally administered substances in mice has indicated that radiolabeled compounds were detected in the lungs and other organs, including the head [26]. Thus, we hypothesize that a portion of the administered 3D8 scFv may bypass the blood–brain barrier, reach the brain and potentially influence its therapeutic effects in the lungs [26,28,29]. Therefore, both the administration route and the engineering of the delivery systems are important factors for enhancing drug efficacy and safety. Additionally, Figure 5 presents a hypothesized mechanism of action for both exogenous and endogenous 3D8 scFv against the influenza virus in vivo. The influenza virus initially targets the upper respiratory tract by entering epithelial cells via endocytosis, subsequently infecting the lower respiratory tract and exacerbating the disease. This infection triggers the production of various cytokines, including IL-6 and TNF-α [30,31,32]. IL-6, critical for the innate and adaptive immune responses against pathogens, plays a crucial role in antiviral immunity, facilitating viral clearance, promoting lung repair, and enhancing survival rates in IL-6-deficient mouse models [21,33]. TNF-α, integral to numerous immunological responses and inflammatory processes, demonstrates antiviral activity against several viruses, including vesicular stomatitis, encephalomyocarditis, herpes simplex, and influenza viruses [34]. Specifically, TNF-α exhibits anti-influenza virus activity as increased viral genome levels were observed in TNF-α deficient mice compared to WT mice [34,35]. In this study, at 4 dpi, the IL-6 and TNF-α gene expression levels were upregulated in the 3D8-treated group; the TG group exhibited higher TNF-α gene expression levels than the H1N1-infected group. Additionally, the expression levels in both the 3D8-treated and TG groups resembled those of the mock group at 20 dpi (Figure 4). During viral infection, TNF-α induces epithelial cells to release various antiviral molecules, such as hydrogen peroxide, defensins, cathelicidins, and azurocidin [35,36,37]. Thus, we believe that along with the antiviral activity of 3D8 scFv, antiviral cytokines, including TNF-α, also controlled the influenza virus. However, some studies showed that the overexpression of IL-6 and TNF-α during influenza virus infection may lead to a cytokine storm, compromising the endothelial barrier, causing lung injury, and exacerbating disease severity [30,32,35,38,39]. Therefore, concerns regarding cytokine storm were raised in the 3D8-treated and TG groups when cytokine gene expression levels exceeded those of the H1N1-infected group. Despite these observations, the overexpression of these cytokines cannot be conclusively proved without a control group specifically induced to experience a cytokine storm. Consequently, further experiments are necessary to investigate comprehensively whether 3D8 scFv induces a cytokine storm.

Taken together, 3D8 scFv treatment via the IN route protected the mice against the influenza A virus, whereas intrinsic 3D8 scFv expression in TG mice also displayed remarkable antiviral activity. In TG mice, 3D8 scFv is constitutively expressed, whereas exogenous treatment is transient and administered post-infection. The higher protection observed in TG mice may therefore reflect their continuous and pre-existing expression of 3D8 scFv rather than inherently greater antiviral activity of endogenous 3D8 scFv. These findings highlight the potential of 3D8 scFv as a promising antiviral agent against IAV. Furthermore, the development of TG models of other infectious diseases would facilitate deeper investigation into its underlying mechanisms of action. In addition, transgenic livestock expressing 3D8 scFv can also be used to control diseases in the future. Finally, the optimization of delivery systems for 3D8 scFv remains essential to enhance its therapeutic applicability in further studies.

## 4. Materials and Methods

### 4.1. Animal Model and Ethics Statement

Seven- to eight-week-old female C57BL/6 mice and six-week-old specific pathogen-free (SPF) BALB/c mice were purchased from DBL company (DBL, Eumseong, Republic of Korea). The animals were housed under controlled temperature and humidity with a 12-h light/dark cycle. During a one-week acclimation period, they had free access to food and water. All animal procedures were approved by the Institutional Animal Care and Use Committee of Sungkyunkwan University (IACUC no.: SKKUIACUC2019-03-07-3).

Transgenic mice expressing 3D8 scFv were generated by Macrogen Co., Seoul, Republic of Korea through standard microinjection techniques. Initially, fertilized eggs were harvested from superovulated C57BL/6NcrjBgi mice. Then, male pronuclei were then injected with a 2.6 kb fragment (4 ng/µL) of 3D8 scFv obtained from the pcDNA3.1/V5-His B (3D8 scFv) vector after digestion with NruI/StuI/PvuI restriction enzymes. These injected eggs were implanted into the oviducts of pseudo-pregnant C57BL/6NcrjBgi recipients. The presence of the transgene was verified in 3-week-old mice by analyzing the genomic DNA with PCR using specific primers (Table S1). The initial transgenic founders (F0 lines) were then bred with wild-type C57BL/6NcrjBgi mice to establish transgenic lines. Subsequent sibling production was done by the breeding program at Macrogen Co. All procedures adhered to the principles of laboratory animal care outlined in NIH publication 85-23 (revised 1986) and were conducted according to the NVRQS guidelines in Korea.

All animals utilized for observing parameter assessment in this study were humanely euthanized at the conclusion of the experiments. Euthanasia was performed under anesthesia induced by intraperitoneal injection of Alfaxan (50 mg/kg) and Rompun (10 mg/kg) on mice weighing 19–21.5 g, followed by cervical dislocation. All experimental procedures were conducted in compliance with the ARRIVE guidelines.

### 4.2. Cell Lines and Viruses

Madin–Darby canine kidney (MDCK) (ATCC CCL-34) cells were maintained in complete Eagle’s minimal essential medium (MEM) (Hyclone, Logan, UT, USA) supplemented with 10% fetal bovine serum (FBS) (Hyclone, USA) and 0.1% antibiotic–antimycotic (ThermoFisher Scientific, Waltham, MA, USA) at 37 °C and 5% CO_2_.

Influenza strains A (IAV)/Puerto Rico/8/1934 H1N1 were propagated in the allantoic fluid of nine-day-old embryonated chicken eggs at 37 °C. The viruses were then collected for three days post-infection and purified using sucrose gradient centrifugation. The viral titer was measured using the plaque assays which were performed on MDCK cells.

### 4.3. Expression and Purification of 3D8 scFv

3D8 scFv gene was cloned into the pIg20-3D8 plasmid and expressed in *Escherichia coli* BL21(De3) pLysE cells by adding 1 mM isopropyl 1-thiol-D-galactopyranoside (IPTG) into Luria–Bertani (LB) broth containing 100 µg/mL ampicillin and 25 µg/mL chloramphenicol for 18 h at 26 °C. The cell culture supernatant was obtained using centrifugation at 6000 rpm for 20 min at 4 °C and filtered through a 0.22 µm filter. Next, 3D8 scFv was purified from the supernatant using an IgG Sepharose 6 fast-flow affinity column (GE Healthcare, Chicago, IL, USA), eluted with acetic acid (0.1 M, pH 3.4), and neutralized with a 0.1 volume of 1 M Tris-HCl (pH 9.0). The purity of the eluted protein was confirmed by sodium dodecyl sulfate–polyacrylamide gel electrophoresis (SDS-PAGE) with Coomassie blue staining. All its key features were checked before using it for further experiments (Figure S1).

### 4.4. Bio Distribution of 3D8 scFv

Mice received 3D8 scFv treatment via intranasal (IN), intravenous (IV), or combined (IN + IV) routes, either as a single dose or repeated doses. Specifically, IV administration was performed three times at 12 h intervals, while IN administration was given three times at 24 h intervals. A volume of 100 μg/injection of 3D8 scFv was administered via IN or IV. IN + IV combined injection was treated with ① 50 μg/injection (total 300 μg) and ② 100 μg/injection (total 600 μg). After 3D8 scFv administration via different routes, mice at different periods (0, 2, 4, 6, and 12 h) were dissected, then 400–500 μL of blood was collected from the abdominal vein, and following by harvesting liver, lungs, and kidneys. These samples were used to do the Western blot. In addition, for immunohistochemistry assay, 4 h after 3D8 scFv injection, 20 mL of PBS was perfused into the left ventricle of the heart to harvest mouse organs (liver, lungs, and kidneys).

In this experiment, eighteen mice were used in each group including IN(100), IN(300), IV(100), IV(300), IN + IV(300), and IN + IV(600). Of these, 15 mice were used to test the retention time of 3D8 scFv, and 3 mice were used to conduct the immunohistochemistry assay. All in vivo experiments were performed under anesthesia (IP injection, Alfaxon 50 mg/kg, Rompun 10 mg/kg).

### 4.5. Plaque Assay

MDCK cells were seeded at 10^6^ cells/well in six-well plates to 90–100% confluency. H1N1 particles were serially diluted 10-fold. The DPBS-washed cells were inoculated with 1 mL of diluted viral suspension and incubated for 1 h at 37 °C. Then, the cells were overlaid with DMEM containing 1% SeaPlaque agarose (Lonza, Walkersville, MD, USA) with 1 μg/mL TPCK. Plaque formation was observed for 3–4 days of incubation for influenza virus using crystal violet staining. Then, plaques were counted, and the percentage of plaque reduction was calculated.

### 4.6. TCID_50_ Assay

MDCK cells were seeded at 4 × 10^4^ cells/well in 96-well plates. After serially diluting the supernatant of the homogenized lung tissues ten-fold, 50 µL diluent was added to the well and incubated at 37 °C for 1 h. Fresh media containing MEM + 0.2% BSA + PSA + 1 μg/mL TPCK was added. The cytopathic effect (CPE) or cell death was observed after three days of incubation. The number of positive and negative wells was recorded, and TCID_50_ was calculated based on the Reed–Muench method.

### 4.7. RNA Extraction and One-Step Quantitative Reverse Transcription PCR (RT-qPCR)

To analyze the 3D8 scFv mRNA expression patterns and viral gene expression levels in TG mice, tissue samples (lung, trachea, spleen, heart, livers, and kidneys) were collected and homogenized. Then, total RNA was isolated using TRI reagent (MRC, Cincinnati, OH, USA) according to the manufacturer’s protocol. A final RNA concentration of 10 ng/μL was used. One-step reverse transcriptase (RT)-qPCR was performed using AccuPower GreenStar RT-qPCR Premix and Master mix (Bioneer, Daejeon, Republic of Korea) and Rotor-Gene Q system (Qiagen, Hilden, Germany) with 50 ng of RNA template. Table S1 lists the primers used to amplify the genes for 3D8 scFv, influenza virus (HA and NA), GAPDH, interleukin 6 (IL-6), and tumor necrosis factor-alpha (TNF-α).

### 4.8. Virus Infection of C57BL/6 and TG Mice and Sample Collection

C57BL/6 and TG mice were challenged intranasally with 30 µL two times 50% lethal dose (2LD_50_) of H1N1/PR8 virus (750 PFU) to ensure that the viral challenge is severe enough to induce significant morbidity and mortality in untreated control group, allowing for a more precise assessment of the therapeutic efficacy of 3D8 scFv. After that, C57BL/6 mice were post-treated with 100 µg of 3D8 scFv/time for three days through the IN route, treatment initiated at 0.5 h post-infection on day 0, followed by administration on days 1 and 2 at 24 h intervals, for a total of three administrations and a cumulative dose of 300 µg per mouse. The clinical signs (survival and weight loss) were monitored daily until day 14. Lung samples were collected for histopathological examination four days postinjection (dpi).

### 4.9. Immunoblot Assay

Examining 3D8 scFv distribution: Total protein was extracted from homogenized liver, lungs, kidneys, and plasma of mice using ProPrep solution (Intron bio) according to the manufacturer’s instructions. Protein concentration was determined using the Bradford assay. 3D8 scFv was analyzed via electrophoresis on a 12% SDS gel and transferred to a PVDF blotting membrane using a semidry method. The membrane was first incubated with polyclonal rabbit anti-3D8 antibody (1:1000 dilution) at 4 °C for 24 h and then with goat anti-rabbit IgG-HRP (A21020, Abbkine, Atlanta, GA, USA) secondary antibody (1:5000 dilution) at 25 °C for 1 h. The target bands were determined via the BrightBAND prestained protein marker (BB0312, Scinomics, Daejeon, Republic of Korea).

Evaluating the HA viral protein level: Total protein was extracted from homogenized mouse lung tissues using ProPrep solution (Intron bio, Seongnam-si, Republic of Korea). The primary antibodies included the polyclonal rabbit anti-HA antibody against IAV (PA5-349291, Invitrogen, Waltham, MA, USA) and antibodies against GAPDH (sc-32233, Santa Cruz Technology, Dallas, TX, USA). After that, the membranes were incubated with goat anti-mouse (G-21040, Invitrogen, USA) and goat anti-rabbit IgG-HRP conjugates (A21020, Abbkine, USA). The membranes were subjected to enhanced chemiluminescence (W3652-050, DawinBio, Hanam, Republic of Korea) and exposed to the film to observe the results. The target bands were determined via the BrightBAND prestained protein marker (BB0312, Scinomics, Daejeon, Republic of Korea).

### 4.10. Histopathology Analysis

Whole-lung samples were collected from mock- and virus-infected, 3D8-treated, and TG mice four dpi. These samples were fixed using 10% neutral-buffered formalin and then embedded in paraffin. The sections were then deparaffinized and stained using hematoxylin and eosin. The stained sections were visualized, and the images were captured using a biological microscope (KB-600, Korea labtech, Osan, Republic of Korea) equipped with Optiview software.

### 4.11. Immunohistochemistry (IHC)

Lung tissues were collected from each group, fixed using 10% neutral buffer formalin, and embedded in paraffin. Then, 5 μm-thick tissue sections were sliced and mounted on slides. After deparaffinizing and rehydrating these sections, antigen retrieval was performed in citrate buffer at 95 °C for 20 min. Then, the sections were washed in TBST (TBS + 0.025% Triton X-100) and blocked in TBST containing 10% FBS and 1% BSA for 2 h. Then, these sections were incubated respectively with polyclonal rabbit anti-3D8 scFv (AbFrontier, Seoul, Republic of Korea) at 4 °C overnight. Then, the samples were incubated respectively with goat anti-rabbit TRITC secondary antibodies (1:1000 dilution) at 25 °C for 1 h. The tissue sections were mounted in an antifade mounting medium, stained with DAPI, and then visualized using a confocal microscope Zeiss LSM 700 (Zeiss, Oberkochen, Gemany).

### 4.12. Statistical Analysis

All data were presented as the mean ± standard deviation (SD) and analyzed using GraphPad Prism version 8 (GraphPad Software, USA). One-way and two-way ANOVA were performed to compare the means between two groups. Differences of * *p* < 0.05, ** *p* < 0.01, *** *p* < 0.001, or **** *p* < 0.0001 were considered significant.

## Data Availability

The authors confirm that the data supporting the findings of this study are available within the article and its Supplementary Materials.

## Supplementary Materials

The following supporting information can be downloaded at: https://www.mdpi.com/article/10.3390/ijms27177850/s1.
