# Supplementary material for: Endogenous Expression of an RNA-Hydrolyzing Minibody Enhances the Therapeutic Effects Against Influenza a Virus Compared to Therapeutically Intranasal Administration
Source: Int J Mol Sci. 2026 Sep 2;27(17):7850. doi: 10.3390/ijms27177850 (PMC13566613; doi:10.3390/ijms27177850)
Supplement: Supplementary file 1 [file ijms-27-07850-s001.zip › ijms-4467189-supplementary.pdf]

Table S1. Primers used in this study for RTqPCR

Primer name	Sequence (5'-3')	Accession No.
**3D8_qRT**	F: ACTGACTTCAGACAAATCCT<br>R: CTGTGACATCACAAGATCTGA	AF232220<br>AF232221
**GAPDH_Ms**	F: GGCATTGCTCTCAATGACAA<br>R: TGTGAGGGAGATGCTCAGTG	NC_000072.7
**H1N1-HA**	F: AGTGCCCAAAATACGTCAGG<br>R: CAGTCCATCCCCCTTCAATA	NC_002017.1
**H1N1-NA**	F: CCGGCAAAGTGATGTGTGTG<br>R: TTGTCACCGAAAACCCCACT	OM488264.1
**IL6**	F: CTTCTTGGGACTGATGCTGGTGAC<br>R: CTGTTGGGAGTGGTATCCTCTGTG	DQ788722.1
**TNFα**	F: CGTCAGCCGATTTGCTATCT<br>R: CGGACTCCGCAAAGTCTAAG	NM_013693.3
**3D8_Detect**	F: CAGAGCTCTCTGGCTAACTAG<br>R: CTGTTGAACAGACTCTGACTG	

Table S2. Serum chemistry result of 3D8 scFv safety test

TEST NAMES	IN-NEG	IN-100	IN-200	IV-NEG	IV-100	IV-200	Unit	REF. RANGE
**TP**	4.6	4.2	4.3	4.4	4.5	4.7	g/dL	4.6-7.3
**ALB**	3.4	3.3	3.6	3.4	3.4	3.6	g/dL	2.6-5.4
**T.Bil**	0.1	0.1	0.2	0.1	0.1	0.1	mg/dL	-
**Glu**	335	295	278	339	303	316	mg/dL	-
**BUN**	33.5	32.2	27.8	33	30.8	33.9	mg/dL	2.0-71
**Creatinine**	0	0	0	0	0	0.1	mg/dL	-
**UA**	3.2	2.2	1.7	2	1.8	2.2	mg/dL	-
**AST**	94	97	155	196	114	126	U/L	46-221
**ALT**	35.1	28.7	30	35	36	46.9	U/L	22-133
**ALP**	147	123	118	131	138	157	U/L	16-200
**γ-GTP**	0	0	0	0	0	0	U/L	-
**LDH**	1411	1795	2193	1904	2092	1510	U/L	-
**CPK**	484	524	1797	1695	1004	2258	U/L	-
**Chol**	106	103	97	100	108	119	mg/dL	34-173
**TG**	233	180	189	124	94	163	mg/dL	16-164
**Amyl**	2652.8	2369.2	2221.5	2280.8	2374.6	2353.5	U/L	-
**Ca**	10.5	9.8	9.3	9.2	9.2	10	mg/dL	-
**Pi**	14.8	15.6	16.3	16.4	15.1	15.6	mg/dL	-
**Lipa**	18	44	45	50	39	41	U/L	-

**Glo**	1.2	0.9	0.7	1	1.1	1.1	g/dL	-
**Na**	152.7	150.6	149.6	149.3	149.8	152.1	mmol/L	153-175
**K**	>10	7.3	7.99	7.88	7.55	8.1	mmol/L	-
**Cl**	112.6	113.3	115.4	114.5	116.7	118.5	mmol/L	-

Serum chemistry test was conducted by Neodin BioVet.Laboratory

Reference range: https://labs.dgsom.ucla.edu/dlam/files/view/docs/diagnostic-lab-services/private/serum_chemistry_reference_ranges_mice.pdf

TP total protein, ALB albumin, T.Bil total bilirubin, Glu glucose, BUN blood urea nitrogen, UA uric acid, AST aspartate aminotransferase, ALT alanine aminotransferase, ALP alkaline phosphatase, LDH lactate dehydrogenase, CPK creatine phosphokinase, Chol cholesterol, TG triglycerides, Amyl amylase, Ca calcium, Pi perfusion index, Lipa lipase, Glo globulin, Na sodium, K potassium, Cl chloride.

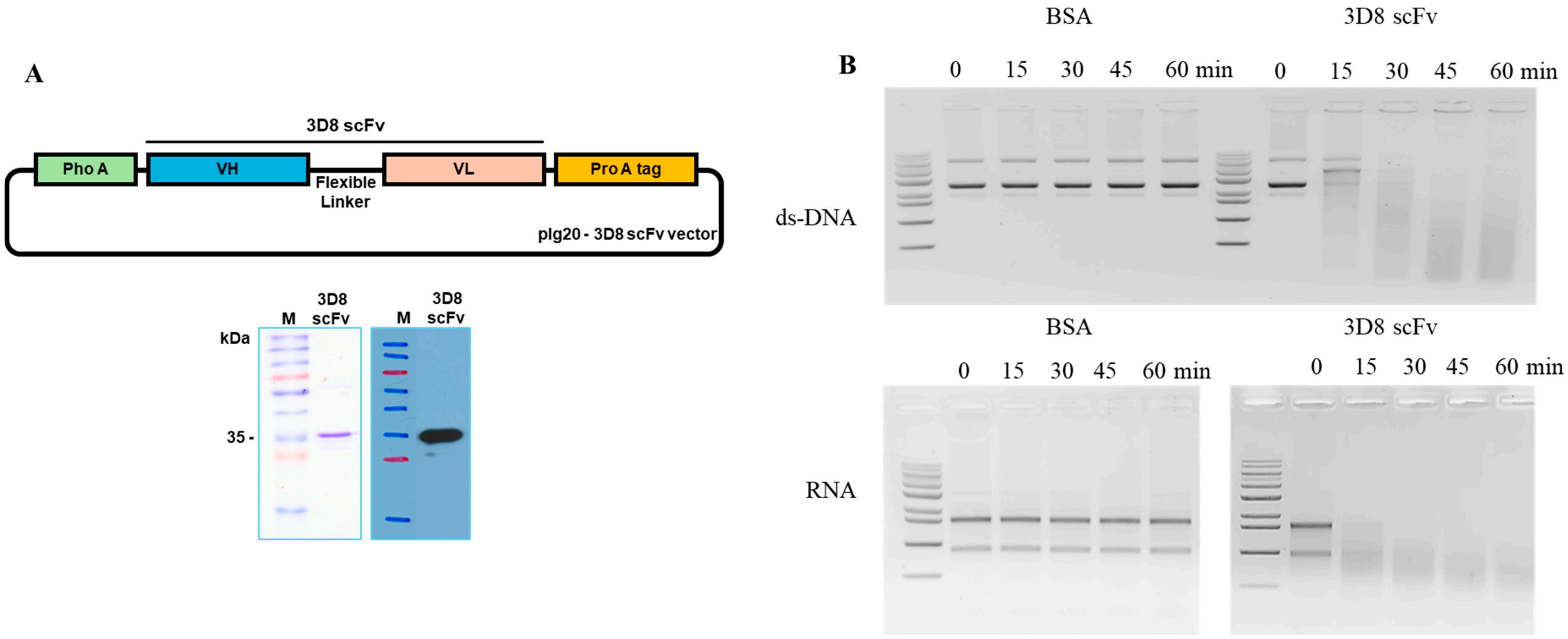

Figure S1. Key characteristics of 3D8 scFv. (A) 3D8 scFv expression plasmid. 3D8 scFv protein was checked using coomassie blue and western blotting. (B) The nucleic acid-hydrolyzing activity was exhibited through dsDNA, and rRNA degradation.

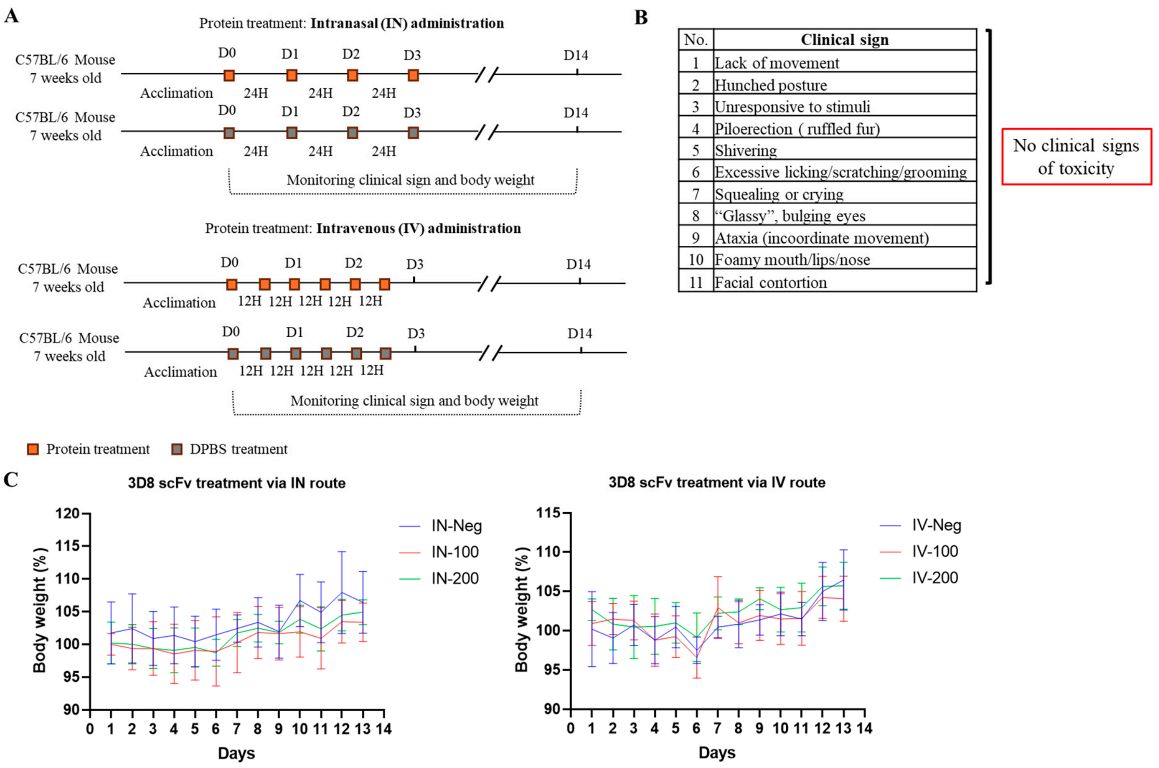

Figure S2. Safety evaluation of 3D8 scFv. (A) Protein treatment scheme: mice were treated with 100 µg/time and 200 µg/time of 3D8 scFv for 4 times - 24H intervals and 6 times – 12H intervals via IN and IV route, respectively. (B) Clinical signs observation daily. (C) The percentage of body weight assessment.
